# Curcumin AntiCancer Studies in Pancreatic Cancer

**DOI:** 10.3390/nu8070433

**Published:** 2016-07-16

**Authors:** Sabrina Bimonte, Antonio Barbieri, Maddalena Leongito, Mauro Piccirillo, Aldo Giudice, Claudia Pivonello, Cristina de Angelis, Vincenza Granata, Raffaele Palaia, Francesco Izzo

**Affiliations:** 1 Division of Abdominal Surgical Oncology, Hepatobiliary Unit, Istituto Nazionale per lo studio e la cura dei Tumori “Fondazione G. Pascale”—IRCCS—Via Mariano Semmola, Naples 80131, Italy; maddalenaleongito@virgilio.it (M.L.); mauropiccirillo73@libero.it (M.P.); r.palaia@istitutotumori.na.it (R.P.); f.izzo@istitutotumori.na.it (F.I.); 2 S.S.D Sperimentazione Animale, Istituto Nazionale per lo studio e la cura dei Tumori “Fondazione G. Pascale”—IRCCS, Naples 80131, Italy; 3 Epidemiology Unit, Istituto Nazionale per lo studio e la cura dei Tumori “Fondazione G. Pascale”—IRCCS—Via Mariano Semmola, Naples 80131, Italy; aldo.giudice@libero.it; 4 Dipartimento di Medicina Clinica e Chirurgia, Sezione di Endocrinologia, Università di Napoli Federico II, Naples 80131, Italy; cpivonello@gmail.com; 5 I.O.S. & Coleman Srl, Naples 80011, Italy; cristinadeangelis83@hotmail.it; 6 Division of Radiology, Istituto Nazionale per lo studio e la cura dei Tumori “Fondazione G. Pascale”—IRCCS—Via Mariano Semmola, Naples 80131, Italy; cinzia.granata80@libero.it

**Keywords:** curcumin, natural compound, pancreatic cancer, therapy

## Abstract

Pancreatic cancer (PC) is one of the deadliest cancers worldwide. Surgical resection remains the only curative therapeutic treatment for this disease, although only the minority of patients can be resected due to late diagnosis. Systemic gemcitabine-based chemotherapy plus nab-paclitaxel are used as the gold-standard therapy for patients with advanced PC; although this treatment is associated with a better overall survival compared to the old treatment, many side effects and poor results are still present. Therefore, new alternative therapies have been considered for treatment of advanced PC. Several preclinical studies have demonstrated that curcumin, a naturally occurring polyphenolic compound, has anticancer effects against different types of cancer, including PC, by modulating many molecular targets. Regarding PC, in vitro studies have shown potent cytotoxic effects of curcumin on different PC cell lines including MiaPaCa-2, Panc-1, AsPC-1, and BxPC-3. In addition, in vivo studies on PC models have shown that the anti-proliferative effects of curcumin are caused by the inhibition of oxidative stress and angiogenesis and are due to the induction of apoptosis. On the basis of these results, several researchers tested the anticancer effects of curcumin in clinical trials, trying to overcome the poor bioavailability of this agent by developing new bioavailable forms of curcumin. In this article, we review the results of pre-clinical and clinical studies on the effects of curcumin in the treatment of PC.

## 1. Introduction

Pancreatic cancer is one of the deadliest cancer worldwide [1]. Surgical resection remains the only curative therapeutic treatment for this disease, although only the minority of patients can be resected due to late diagnosis [2]. Systemic gemcitabine-based chemotherapy has been used as the standard therapy for patients with advanced PC, although this treatment is associated with many side effects and poor overall survival [3,4]. In order to improve the overall survival of patients with PC, many studies combined the use of gemcitabine with different agents, although the results were not encouraging [5,6,7,8,9,10,11]. For these reasons, new alternative therapies involving natural compounds with minimal toxicity, such as curcumin, have been considered for treatment of PC. Curcumin, a naturally occurring polyphenolic compound, derives from turmeric (*Curcuma longa*). It has been commonly used as a food additive or dietary pigment and in traditional medicine [12,13,14,15,16]. Preclinical in vitro and in vivo studies have demonstrated that curcumin has several pharmacologic effects, including antioxidant, anti-inflammatory, and anticancer activities, in different types of cancer, including PC, by modulating multiple signaling pathways [15,17,18,19,20,21,22,23,24,25,26,27,28,29,30,31,32,33,34,35,36,37,38,39,40,41,42,43,44]. Taken together, these results suggest that curcumin can be considered a new therapeutic drug in PC treatment [45]. In addition it has many advantages for patients, such as safety and minimal toxicity. Several researchers tested the anticancer effects of curcumin in clinical trials, trying to overcome the poor bioavailability of this agent by developing new bioavailable forms of curcumin [15,46,47,48,49,50,51,52,53,54,55,56,57]. In this article, we review the results of pre-clinical and clinical studies on the effects of curcumin in the treatment of pancreatic cancer. 

## 2. Effects of Curcumin in Treatment of PC

*(a)*  In Vitro Studies: Dissecting the Molecular Mechanism Underlying the Antitumor Effects of Curcumin in PC Cell Growth

Several preclinical studies showed that curcumin has antitumor effects by modulating multiple cell-signaling pathways in different types of cancers, including colorectal [28,33,58], pancreatic [17,18,22,27,28,29,30,31,34,35,42,43,59,60,61,62,63,64,65,66,67], breast [26], lung [32], hepatic [20], ovarian [25], head and neck [68], and prostate [24].

Regarding PC, in vitro studies on the effects of curcumin have been performed on different PC cells lines including MiaPaCa-2, MPanc-96, BxPC-3, Panc-1, AsPC-1, and L3.6pL. Results from these studies showed that the anti-proliferative effects of curcumin are mainly due to the inhibition of oxidative stress and angiogenesis and the induction of apoptosis [17,18,22,29,34,42,43,59,60,65,69,70,71,72,73]. The first report on the antitumor effect of curcumin in PC was described by Li et al. [17]. The authors demonstrated that curcumin down-regulated Nuclear factor kappa-light-chain-enhancer of activated B cells (NF-κB) and growth control molecules induced by NF-κB in human pancreatic cells in a time- and dose-dependent manner. These effects were accompanied by marked growth inhibition and apoptosis. Similar results were obtained by Wang et al. [22]. Then authors demonstrated that the Notch-1 signaling pathway was associated with NF-κB activity during curcumin-induced cell growth inhibition and apoptosis of pancreatic cells, suggesting that the down-regulation of Notch signaling by curcumin could represent a novel strategy for the treatment of patients with PC. In another study, it was demonstrated that curcumin treatment inhibited the proliferation of BxPC-3 human pancreatic cancer cells by DNA damage-mediated G2/M cell cycle arrest, by inhibition of cyclin B1/Cyclin-dependent kinase 1 (Cdk1) expression and by the activation of ataxia tel-angiectasia mutated (ATM)/Checkpoint kinase 1(Chk1)/Cell Division Cycle 25C (Cdc25C) [29]. Jutooru et al. showed that curcumin inhibited NF-κB expression and Panc-1 and L3.6pL cancer cell growth by down-regulation of the specificity protein Sp1 [59]. We also demonstrated that curcumin inhibited the proliferation and enhanced the apoptosis of MIA PaCa-2 cells, through the suppression of NF-κB-activation [18]. Recent findings showed that curcumin induced apoptosis in PC cells through the induction of forkhead box O1 (FOXO1) and the inhibition of the phosphatidylinositol 3-kinase/phosphatidylinositol 3-kinase (PI3K/Akt) pathway [43]. The antitumor role of curcumin in PC was also demonstrated by Diaz et al. in Panc-1 cells. The authors showed that curcumin induced pancreatic adenocarcinoma cell death via the reduction of the inhibitors of apoptosis (IAP) [42]. Finally, very recently, it was demonstrated that a small-molecule tolfenamic acid and dietary spice curcumin treatment enhanced the anti-proliferative effect in PC cells L3.6pl and MIA PaCa-2 through Sp1 suppression, NF-κB disruption of translocation to the nucleus and cell cycle phase distribution [34].

**Figure 1:**
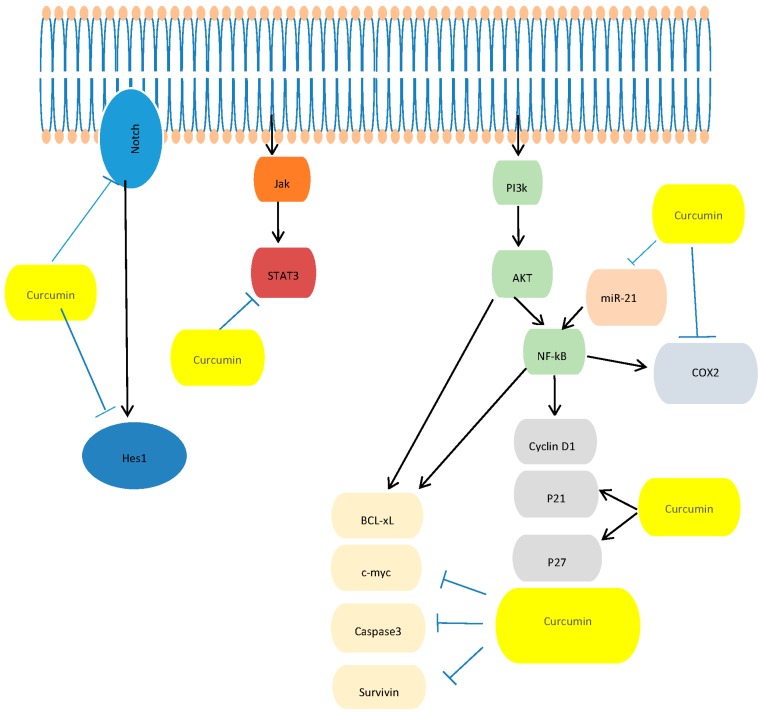
A schematization of molecular targets in PC regulated by curcumin. NF-κB: Nuclear factor kappa-light-chain-enhancer of activated B cells; COX2: Cyclooxygenase 2; Hes-1: Cyclin-dependent kinase 1; Akt: Protein kinase B; Stat3: Signal transducer and activator of transcription 3; PI3K: phosphatidylinositol 3-kinase; Notch-1: Neurogenic locus notch homolog protein-1; c-myc: C-mycproto-oncogene; Jak: Janus kinase. P21: Cyclin-dependent kinase inhibitor; P27: Cyclin-dependent kinase inhibitor; BCL-xL: B-cell lymphoma-extra large.

These results suggest that curcumin exerts its antitumor effect on PC by acting on different molecular mechanisms. Specifically, other studies showed that treatment of PC cells with curcumin has been associated with reduced migration and invasiveness of tumor cells, inhibition of cancer stem cell function, reversal of the epithelial-mesenchymal transition (EMT), and suppression of miR-221, Cyclooxygenase 2 (COX-2) and their effectors and pro-inflammatory cytokines [69,70]. In addition, it has been demonstrated that curcumin can also block signal transducer and activator of transcription 1 (STAT1) and signal transducer and activator of transcription 3 (STAT3) phosphorylation, and epidermal growth factor receptor (EGFR) and (neurogenic locus notch homolog protein-1) Notch-1 signaling pathways, which play important roles in pancreatic tumor growth [74]. It has been also demonstrated that siRNA/shRNA, small-molecule kinase inhibitors, and curcumin targeting these tumor stem cell markers and tumor suppressor miRNAs could be the perfect therapeutic agents for the treatment of PC [31,67,69,75,76,77] (Figure 1).

In order to ameliorate the aqueous solubility of curcumin, different derivatives of this compound or delivery system have been developed [78,79]. One curcumin analogue used in in vitro experiments is the 3,4-difluorobenzylidene curcumin (CDF). This compound has a higher tendency to accumulate in the pancreas than normal curcumin [74,80]. Although it has been demonstrated that CDF has cytotoxic effects on both resistant and nonresistant pancreatic tumor cell lines with respect to curcumin, this curcumin derivative still presents low aqueous solubility. To bypass this problem, researchers developed a new delivery system based on nanoparticles, such as hyaluronic acid (HA)-conjugated polyamidoamine dendrimers and hyaluronic acid (HA) and styrene-maleic acid-engineered nanomicelles of CDF. Results from studies performed with these systems gained improvements of aqueous solubility, stability, release profile and antitumor effects on PC cells lines with respect to unformulated CDF [56,63,81]. Table 1 summarizes the most relevant in vitro studies on the antitumor effect of curcumin in PC cells.

*(b)*  In Vivo Studies: Effects of Curcumin in Mouse Model of PC

The antitumor effect of curcumin and its analogues on PC has been demonstrated in in vivo experiments on mouse models of PC [18,27,59,71,74,82,83,84,85,86,87,88]. The first in vivo study was reported by Kunnumakara et al. [71]. The authors demonstrated that curcumin (1 g/kg orally) potentiated the antitumor activity of gemcitabine (25 mg/kg via intraperitoneal injection) in an orthotopic mouse model of PC [15] through the suppression of proliferation, angiogenesis, and inhibition of NF-κB-regulated gene products. Our research group also reported similar results. In fact, we demonstrated with the generation of an orthotropic mouse model of PC that tumors from mice injected with MIA PaCa-2 cells and placed on a diet containing curcumin at 0.6% for six weeks were smaller than those observed in controls. We also showed a down-regulation of the NF-κB-regulated gene products, suggesting that curcumin had great potential in the treatment of human PC, through the modulation of the NF-κB pathway [18]. Mach et al., in a xenograft human PC model, established the minimum effective dose (MED, 20 mg/kg) and optimal dosing schedule for liposomal curcumin [88]. In another study, the in vivo antitumorigenic activity of curcumin was investigated in athymic nude bearing L36pL cells as xenografts. The authors demonstrated that curcumin (dose of 100 mg/kg/days) inhibited tumor growth and tumor weight by down-regulation of the Sp transcription factor [59]. In order to potentiate the effects of curcumin on PC in vivo, several studies were performed using different forms of curcumin. Bao et al. demonstrated that CDF (2.5 mg/mouse/days; 5 mg/mouse/days; intragastric once daily for three weeks), an analogue of curcumin analogue, inhibited pancreatic tumor growth by switching on suppressor microRNAs and attenuating the expression of histone methyltransferase enhancer of zeste homolog 2, EZH2 [74]. Similar effects were reported for synthetic curcumin analogues EF31 and UBS109. The authors demonstrated, both in vitro and in vivo, that these analogues were potent DNA hypomethylating agents in PC [86]. The efficacy of liposomal curcumin in human PC was also reported by Ranjan et al. The authors showed that in xenograft tumors in nude mice, liposomal curcumin (20 mg/kg i.p. three times a week for four weeks) induced a suppression of tumor growth compared to untreated controls, indicating that this agent could be beneficial in patients with PC [85]. Similar results have been reported by recent studies in which the efficacy of curcuminoids and nanomicelles in treatment of PC was demonstrated [81,82]. It is important to underline that in all studies performed with curcumin derivatives and or a delivery system, the antitumor effects have been reported to be greater with respect to those observed with conventional curcumin. On the basis of these results, researchers tested the anticancer effects of curcumin in clinical trials, trying to overcome the poor bioavailability of this agent by developing new bioavailable forms of curcumin. Table 2 summarizes preclinical in vivo studies on the anticancer effects of curcumin against PC.

*(c)*  Clinical Trials

In order to translate the preclinical antitumor effects of curcumin into clinical practice, few clinical trials have been performed so far. Healthy volunteers and cancer patients were treated with curcumin, administered orally, in different clinical trials (phase I and pharmacokinetic studies). No dose-limiting toxicity (DLT) of up to at least 12 g/day was observed in patients, although nausea and diarrhea have been reported [48,89,90]. It was established that the daily oral dose of curcumin of 8 g or less is the most commonly used in clinical trials, due to its poor bioavailability.

Several phase II clinical trials on the antitumor effects of curcumin in PC were conducted [91,92,93]. Dhillon et al. conducted the first trial [91] and successfully tested the safety and the efficacy of curcumin used as a monotherapy in 25 patients of PC. Another group conducted a phase I/II clinical trial of curcumin in 21 patients with PC (resistant to gemcitabine-based chemotherapy), combining gemcitabine-based chemotherapy with curcumin treatment (8 g daily oral dose) [92]. Results from this study indicated that combination therapy using 8 g oral curcumin daily with gemcitabine-based chemotherapy was safe and feasible in patients with PC. Another interesting study tested the efficacy and feasibility of curcumin (8 g daily oral dose) in combination with gemcitabine monotherapy (standard dose and schedule) in 17 chemo-naive patients with PC. Differently from previous studies, increased gastrointestinal toxicity was observed in seven patients treated with this therapy, probably due to the elevated dose of curcumin combined with gemcitabine. For this reason, the dose of curcumin was reduced from 8 to 4 g [93]. From this study emerged the problem of the poor bioavailability of curcumin, which strongly limited its application in clinical practice. To solve this problem, new curcumin analogs and new drug delivery systems have been developed [46,47,48,49,50,51,52,53,54,55]. Interesting results have been reported by dose escalation and pharmacokinetic studies performed with Theracurcumin, a nanoparticle-based curcumin [55]. These studies demonstrated that the plasma curcumin levels observed after Theracurcumin ingestion were higher with respect to those obtained with conventional curcumin. The phase I clinical trial involving Theracurcumin (level 1 group: 200 mg oral/daily; level 2 group: 400 mg oral/daily) was conducted on 16 patients with PC resistant to gemcitabine-based chemotherapy [94]. The results from this study showed that repetitive systemic exposure to high concentrations of Theracurmin did not increase the incidence of side effects in cancer patients receiving gemcitabine-based chemotherapy, indicating that this agent could represent a new agent for PC treatments.

New clinical trials are needed to test the therapeutic effects of curcumin and its analogues in patients with PC.

## 3. Conclusions

Several preclinical studies have demonstrated that curcumin, a naturally occurring polyphenolic compound, has anticancer effects against different types of cancer, including PC, by modulating many molecular targets. On the basis of these results, several researchers tested the anticancer effects of curcumin in clinical trials, trying to overcome the poor bioavailability of this agent, which limited its clinical application. New bioavailable forms of curcumin have been developed and the results from clinical trials on patients with PC suggest that these agents could represent promising new treatments for PC, although more clinical studies will be still needed.

## Figures and Tables

**Table 1:** A summary of in vitro studies on the role of curcumin in Pancreatic Cancer cell growth.

Cell Lines	Dose of Curcumin (μM)	Molecular Targets	Reference
MiaPaCa-2; BxPC-3; Panc-1; MPanc-96	≥25	NF-κB↓;VEGF↓	[71]
MiaPaCa-2	50	NF-κB↓	[18]
BxPC-3	2.5	Cdk1↓; cyclin B1↓	[29]
Panc-28; L3.6p	≥25	NF-κB↓, Sp-1, Sp-3, Sp4↓	[59]
Miapaca-E; Miapaca-M; BxPC-3	≥4	miR-220↑; miR-21↓	[31]
Panc-1	≥25	IAP↓	[42]
L3.6pl; MIA PaCa-2	5–25	NF-κB↓, Sp-1, Sp-3, Sp4↓	[34]
PANC-1	10–30	Shh↓, GLI1↓, E-cadherin↓, vimentin↓	[70]

NF-κB: Nuclear factor kappa-light-chain-enhancer of activated B cells; VEGF: Vascular endothelial growth factor; Cdk1: Cyclin-dependent kinase 1; Sp-1: Specificity protein 1; Sp-3: Specificity protein 3; Sp4: Specificity protein 4; IAP: inhibitors of apoptosis; Shh: Sonic hedgehog, GLI1: Glioma-associated oncogene homologue 1; E-cadherin: Epithelial cadherin.

**Table 2:** Preclinical in vivo studies on the anticancer effects of curcumin against PC.

Animal Models	Drug	Dose of Curcumin	Effects	Reference
Orthotopic mouse model (MIA PaCa-2 cells)	Curcumin + Gemcitabine	1 g/kg (orally)	Suppression of proliferation, angiogenesis, and inhibition of NF-κB in tumors	[71]
Orthotopic mouse model (MIA PaCa-2 cells)	Curcumin	0.6% for 6 weeks (dietary food)	Tumor growth inhibition and down regulation of the NF-κB-regulated gene products	[18]
Xenograft mouse model (L36pL cells)	Curcumin	100 mg/kg/days	Tumor growth and Tumor weight inhibition	[59]
Orthotopic mouse model (MIA PaCa-2 cells)	CDF	2.5 mg/mouse/days; 5 mg/mouse/days; intragastric once daily for 3 weeks	Tumor growth inhibition, reduced expression of EZH2	[74]
Xenograft mouse model (MIA PaCa-2 cells)	Liposomal curcumin	20 mg/kg i.p. three-times a week for four weeks	Tumor growth inhibition	[85]

## Abbreviations

PC	Pancreatic cancer
NF-κB	Nuclear factor kappa-light-chain-enhancer of activated B cells
Cdk1	Cyclin-dependent kinase 1
ATM	Ataxia tel-angiectasia
Chk1	Checkpoint kinase 1
Cdc25C	Cell division cycle 25C
FOXO1	Forkhead box O1
PI3K	Phosphatidylinositol 3-kinase
Akt	Phosphatidylinositol 3-kinase
Sp1	Specificity protein
IAP	Inhibitors of apoptosis
EMT	epithelial-mesenchymal transition
STAT1	Activator of transcription 1
STAT3	Signal transducer and activator of transcription 3
EGFR	Epidermal growth factor receptor
Notch-1	Neurogenic locus notch homolog protein-1
COX2	Cyclooxygenase 2
Hes-1	Cyclin-dependent kinase 1
Akt	Protein kinase B
Stat3	Signal transducer and activator of transcription 3
PI3K	phosphatidylinositol 3-kinase
Notch-1	Neurogenic locus notch homolog protein-1
c-myc	C-mycproto-oncogene
Jak	Janus kinase
P21	Cyclin-dependent kinase inhibitor
P27	Cyclin-dependent kinase inhibitor
BCL-xL	B-cell lymphoma-extra large
CDF	3, 4-difluorobenzylidene curcumin
HA	hyaluronic acid
Sp-3	Specificity protein 3
Sp4	Specificity protein 4
Shh	Sonic hedgehog
GLI1	Glioma-associated oncogene homologue 1
E-cadherin	Epithelial cadherin
EZH2	enhancer of zeste homolog 2
MED	minimum effective dose
DLT	dose-limiting toxicity
